# Comparison of Transoral and Transcervical Ultrasonography with MRI for the Diagnostic Work-Up of Oropharynx Tumors: A Protocol for a Multicenter Clinical Trial (SPOTUS)

**DOI:** 10.3390/diagnostics14060577

**Published:** 2024-03-08

**Authors:** Martin Garset-Zamani, Gitte Bjørn Hvilsom, Thomas Kjærgaard, Christina Caroline Plaschke, Christoffer Holst Hahn, Mikkel Kaltoft, Padraig O’Leary, Natalie Lassen Frid, Rikke Norling, Danijela Dejanovic, Johanna Maria Hall, Tina Klitmøller Agander, Signe Bergliot Nielsen, Annette Kjær Ersbøll, Irene Wessel, Christian von Buchwald, Tobias Todsen

**Affiliations:** 1 Department of Otorhinolaryngology, Head and Neck Surgery and Audiology, Copenhagen University Hospital—Rigshospitalet, 2100 Copenhagen, Denmarktobias.todsen@regionh.dk (T.T.); 2 Institute of Clinical Medicine, Faculty of Health and Medical Sciences, University of Copenhagen, 2200 Copenhagen, Denmark; 3 Department of Otorhinolaryngology, Head and Neck Surgery, Zealand University Hospital, 4600 Køge, Denmark; 4 Department of Otorhinolaryngology, Head and Neck Surgery & Audiology, Aarhus University Hospital, 8200 Aarhus, Denmark; 5 Department of Radiology, Copenhagen University Hospital—Rigshospitalet, 2100 Copenhagen, Denmark; 6 Department of Clinical Physiology and Nuclear Medicine, Copenhagen University Hospital—Rigshospitalet, 2100 Copenhagen, Denmark; 7 Department of Pathology, Copenhagen University Hospital—Rigshospitalet, 2100 Copenhagen, Denmark; 8 Copenhagen Emergency Medical Services, University of Copenhagen, 2100 Copenhagen, Denmark; 9 National Institute of Public Health, University of Southern Denmark, 2100 Copenhagen, Denmark; 10 Copenhagen Academy for Medical Education and Simulation, The Capital Region of Denmark, 2100 Copenhagen, Denmark

**Keywords:** oropharyngeal cancer, palatine tonsil, lingual tonsil, base of tongue, tonsil cancer, oropharyngeal squamous cell carcinoma, human papillomavirus, transcervical ultrasound, transoral ultrasound, intraoral ultrasound

## Abstract

This study protocol for a prospective, multicenter, diagnostic, clinical trial describes the integration of transoral and transcervical ultrasonography (US) in the initial clinical work-up of patients referred to tertiary head and neck cancer centers with suspected oropharyngeal cancer. The study evaluates the blinded detection rate of oropharyngeal tumors and their US-estimated size and T-stage before histopathology and cross-sectional imaging are available. Magnetic resonance imaging (MRI) scans will be prospectively rated while blinded to T-site histopathology and US. The primary outcome measures of diagnostic accuracy, including sensitivity, specificity, positive and negative predictive values, and overall accuracy, will be reported for both US and MRI. A sub-analysis of prospectively rated 18F-Fluorodeoxyglucose (FDG) positron emission tomography/computerized tomography (PET/CT) scans in patients with clinically suspected unknown primary tumors will also be compared to US and MRI. Secondary outcome measures, including a comparison of tumor size estimation between US, MRI, and CT, will also be reported. This prospective multicenter study will provide clinically impactful information regarding the use of transoral and transcervical US for the diagnostic work-up of oropharyngeal cancer.

## 1. Introduction

The incidence of oropharyngeal squamous cell carcinoma (OPSCC) has increased in Western countries during the past few decades [1,2,3] due to human papillomavirus (HPV) [4,5,6,7]. HPV-associated OPSCCs often present with metastases in the neck, despite small primary tumors [2,8]. Detection of the primary tumor by clinical examination is challenging due to the small size, which causes the tumor to be visually obscured or impalpable. Accordingly, these patients require multiple diagnostic imaging modalities or surgery to locate the primary tumor [9,10,11]. Magnetic resonance imaging (MRI) is regarded as the best soft-tissue imaging modality for OPSCCs. It offers visualization of the entire oropharynx, but resolution is limited when differentiating smaller focal tumors from unilateral hypertrophic tonsil tissue [12]. 18F-Fluorodeoxyglucose (FDG) positron emission tomography/computerized tomography (PET/CT) further improves the detection rate of smaller OPSCCs, but physiologic asymmetry in FDG-uptake of oropharyngeal lymphoid tissue can mimic the FDG-uptake of primary tumors [13,14,15]. 

Two studies have previously compared MRI, PET/CT, and CT scans with transcervical ultrasonography (US) to detect OPSCCs [16,17]. While not performed blinded to histopathology, the studies found high detection rates of 90–98% using transcervical US compared to 71% (MRI), 69–83% (CT), and 83% (PET/CT). For small or unknown primary tumors, the literature is currently limited to smaller pilot studies or case reports [18,19]. The optimization of US transducers for the visualization of deep-seated oropharyngeal structures require sacrificing image resolution for increased depth penetration, which may diminish the visibility of smaller tumors [20]. Instead, high-frequency US (>15 MHz) provides a higher-resolution image of superficial structures [21]. With the development of small-footprint US transducers, the intraoral US technique has shown potential for oral tongue cancer detection and depth-of-invasion estimation [22,23]. For the oropharynx, a transoral US approach has shown improved visualization compared to transcervical US for the diagnosis of peritonsillar abscesses [24,25]. A small-sample feasibility study found that outpatient-performed transoral US had high sensitivity and specificity to differentiate oropharyngeal tumors from normal tissue, but the study was not performed blinded to histopathology for all included cases [26]. Prospective multicenter trials blinded to cross-sectional imaging and histopathology are required to establish the diagnostic accuracy of transoral and transcervical US for oropharyngeal tumor detection and staging. 

### Research Question

In first-time patient referrals to tertiary head and neck cancer centers with suspicion of oropharyngeal cancer, what is the diagnostic accuracy of outpatient-performed transoral and transcervical US compared to MRI for tumor detection and staging?

## 2. Materials and Methods

### 2.1. Study Design and Setting

We will conduct a prospective, multicenter, clinical trial exploring the use of transoral and transcervical US in the outpatient clinic for detecting oropharyngeal tumors, blinded to reference standard imaging and histopathology (Clinicaltrials.gov registration NCT05696314). The Surgeon-Performed Oropharyngeal Transoral UltraSonography (SPOT-US) trial will be a single-group paired diagnostic study comparing transoral and transcervical US to MRI and PET/CT [27]. The aim of the trial is to explore whether US can improve the current diagnostic work-up of OPSCCs. 

### 2.2. Participant Eligibility

The trial will be performed at the department of Otorhinolaryngology, Head and Neck Surgery in three public hospitals in Denmark: Copenhagen University Hospital—Rigshospitalet, Aarhus University Hospital, and Zealand University Hospital, Køge. In Denmark, patients suspected of having cancer are entitled to a free diagnostic work-up and treatment at public hospitals, funded by the national tax system.

Adult patients aged 18+ years will be screened for eligibility prior to their first clinical work-up at the departments by a study investigator at each center and registered in a screening log (Table 1). 

During examination at the outpatient clinic in the above-mentioned hospital departments, potentially eligible patients will be assessed for eligibility by a head and neck surgeon or study investigator (Table 2). If a potentially eligible patient is unable to be included due to unavailability of study investigators, the baseline characteristics (initial clinical diagnosis, age, and sex) and the final diagnosis (tumor type, location, and T-stage) will be compared with included patients to assess for potential selection bias (Table 1).

### 2.3. Outpatient-Performed Clinical Exam with Transoral and Transcervical US (Index Test)

#### 2.3.1. Standard Clinical Work-Up

Patients with suspected head and neck cancer enter a fast-track outpatient clinic in tax-funded public hospitals’ head and neck surgery departments within one week from referral. The standard Danish oropharyngeal cancer work-up and proposed study interventions are summarized in Table 3. Clinical examinations are performed by otorhinolaryngology senior registrars or consultants. 

#### 2.3.2. Prospective Clinical Data Collection during Initial Outpatient Clinic Exam

We will prospectively register oropharyngeal findings from inspection and digital endoscopy (no visible pathology, visible tumor, or visible asymmetry), palpation (palpable tumor or no palpable findings), narrow-band imaging (suspect vasculature or normal vasculature), tumor suspicion (highly suspected tumor, inconclusive asymmetry, or no tumor detected), tumor location (palatine tonsil, lingual tonsil, overlapping palatine and lingual tonsil regions, or other oropharyngeal sites), tumor size in three diameters, tumor invasion of underlying tissue (yes, no, or inconclusive), and Union for International Cancer Control 8th Edition (UICC8) clinical T-stage (Tx, T0, T1, T2, T3, T4) [28]. Patient-reported discomfort from oropharyngeal palpation will be rated on an 11-point numeric rating scale from 0 (no discomfort) to 10 (worst discomfort imaginable) [29]. 

**Table 3:** Standard oropharyngeal cancer work-up in Denmark and integration of the proposed study interventions.

Setting	Standard Diagnostic Work-Up	Study Interventions
Otorhinolaryngology, Head and Neck Surgery outpatient clinic (Initial work-up)	Clinical exam including inspection, palpation, and digital endoscopy with narrowband imaging. Surgeon-performed neck ultrasound [30]. Fine-needle aspiration cytology. Biopsies from T-site.	Transoral ultrasound of the oropharynx. Transcervical ultrasound of the oropharynx. Optional ultrasound-guided needle biopsy from the oropharynx.
Diagnostic imaging	Head and neck MRI. Chest CT. ° PET/CT. *	
Diagnostic operations	Direct laryngoscopy with frozen-section biopsy. ^§^ Palatine tonsillectomy. * Lingual tonsillectomy. *	
MDT conference and treatment-initiation planning	Final histopathological diagnosis, tumor location, and UICC8 TNM-stage established. Treatment modality chosen: surgical, (chemo)radiotherapy, or palliative treatment.	

° Chest CT is included to evaluate potential distant metastases to the lungs per Danish national guidelines for oropharynx cancer [31]. * These procedures are additional procedures performed in patients suspected of having head and neck cancer of unknown primary. In Denmark, diagnostic lingual tonsillectomy is performed as a transoral robot-assisted surgery for HPV-associated cancer of unknown primary if the primary tumor has not been found during direct laryngoscopy and palatine tonsillectomy [11]. ^§^ This procedure is performed if the primary tumor cannot be located or reached in the outpatient clinic.

The following data will be gathered prospectively from medical charts: tobacco smoking, alcohol consumption, multidisciplinary team (MDT) diagnosis and tumor location, cross-sectional imaging data (including MRI, CT, and PET/CT), number of lymph node metastases (solitary or multiple), type of lymph node metastases (cystic or solid), histopathology results from biopsies or tumor resections, and HPV results (p16 status, HPV status, and HPV genotype, described previously) [2]. All data will be recorded in a REDCap database [32]. These data will be used for demographics and descriptive statistics.

#### 2.3.3. Transoral and Transcervical US Training

Prior to initiating inclusion, all participating study investigators performing transoral and transcervical US will complete an introductory written test containing questions on important anatomical landmarks of the oropharynx using transoral or transcervical US, followed by practical performance of the scanning procedures under observation from the primary study investigator (M.G.-Z.). For all transoral US exams, study investigators will follow the systematic scanning approach described in this protocol, while the transcervical US exam is inspired by Coquia et al. [20].

#### 2.3.4. Outpatient-Performed Transoral US

**Figure 1:**
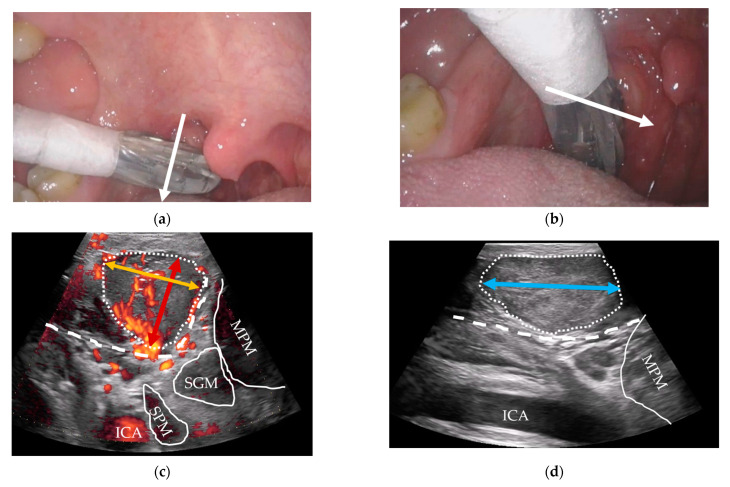
Transoral US of the palatine tonsils: (**a**) a sheathed US transducer placed on the right palatine tonsil (arrow: swipe direction cranial-to-caudal) in the transverse plane; (**b**) sagittal plane (arrow: swipe direction lateral-to-medial); (**c**) transverse US image with Power Doppler of a right palatine tonsil tumor (dotted outline) with underlying constrictor muscle (dashed line), internal carotid artery (ICA), stylopharyngeus muscle (SPM), styloglossus muscle (SGM), and medial pterygoid muscle (MPM). The anteroposterior (red bi-directional arrow) and mediolateral (orange bi-directional arrow) tumor diameters are shown; (**d**) sagittal US image of the same tumor (dotted outline) with the underlying constrictor (dashed line), ICA, and MPM. In sagittal view, the MPM can be used as a craniolateral landmark. The craniocaudal diameter of the tonsil is shown using a blue bi-directional arrow.

**Figure 2:**
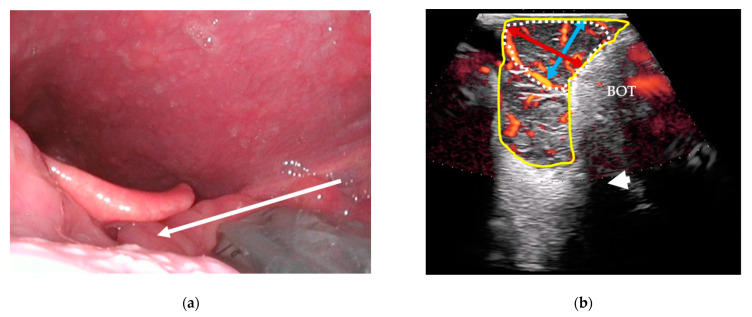
Transoral US of the lingual tonsils: (**a**) a sheathed US transducer placed on the left lingual tonsil (arrow: swipe direction lateral-to-midline) in the sagittal plane; (**b**) sagittal US image with Power Doppler of the lingual tonsil (yellow outline), a tumor-suspected area (dotted outline), underlying base of tongue musculature (BOT), and the hyoid bone’s acoustic shadow (white arrowhead). The craniocaudal (blue bi-directional arrow) and anteroposterior (red bi-directional arrow) diameters are shown on the tumor-suspected area.

Following clinical examination, included patients will be offered combined transoral and transcervical US of the oropharynx during the initial work-up as an extension of the clinical work-up. This will be performed prior to any available T-site histopathology results. US will be performed blinded to any cross-sectional imaging. To perform the transoral US, a patient seated upright in an examination chair will receive topical anesthetic spray applied to both palatine tonsils and the posterior portion of the tongue using xylocaine (10 mg/dose). Patients are informed that eating or drinking following local anesthesia should be postponed for up to one hour due to the risk of aspiration. Patients are instructed to swallow the anesthesia. A sheathed or disinfected “hockey-stick” or small-footprint US transducer is advanced into the patient’s mouth and placed on top of the suspected palatine tonsil in the transverse plane (Figure 1a,c) with a gentle swiping motion from the cranial to caudal pole. The transducer is rotated to the sagittal plane by positioning the tip of the transducer caudally (Figure 1b,d) and a swipe is performed from the lateral to the medial edge. The procedure is repeated contralaterally. Power Doppler is performed on both palatine tonsils. If a lingual tonsil tumor is suspected, a transoral US is attempted (Figure 2). 

We will register additional clinical parameters that may affect incomplete transoral US scans such as mouth opening ability (measured in millimeters between incisors) using a measuring device such as the TheraBite^®^ and the visibility of oropharyngeal structures (graded using the Mallampati scoring system) [33], and objectively rate the patients’ gagging severity (1—Normal; 2—Mild; 3—Moderate; 4—Severe; 5—Very severe) using the Gagging Severity Index [34]. Incomplete transoral US examination will be registered with one or more of the above-mentioned reasons.

#### 2.3.5. Outpatient-Performed Transcervical US

**Figure 3:**
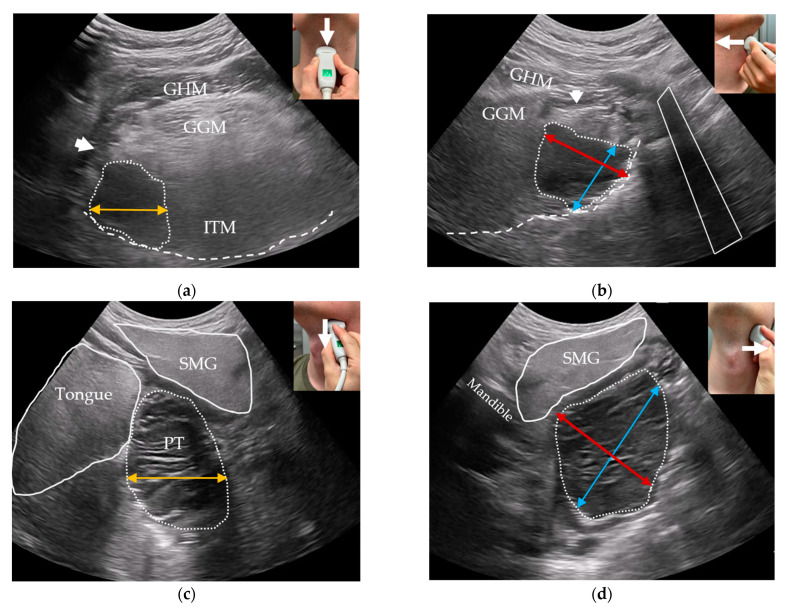
Transcervical US of the palatine and lingual tonsils. Transducer orientations are represented on the top right of each image with a white arrow representing the swiping directions: (**a**) oblique transverse US image of the base of tongue with the geniohyoid (GHM), genioglossus muscles (GGMs), intrinsic tongue muscles (ITMs), hyoglossus muscle (arrowhead), oral surface of the tongue (dashed line), and a hypoechoic endophytic tumor in the right base of tongue (dotted outline). The mediolateral diameter of the tumor is shown (orange bi-directional arrow); (**b**) sagittal US image of the same tumor with the hyoid bone’s acoustic shadow shown (white outline)—craniocaudal (blue bi-directional arrow) and anteroposterior (red bi-directional arrow) tumor diameters; (**c**) Oblique transverse US image of a benign hyperplastic palatine tonsil (PT, dotted outline), the submandibular gland (SMG), tongue, and mediolateral diameter of the PT (orange bi-directional arrow); (**d**) sagittal US image of the same PT with the mandible’s acoustic shadow shown anteriorly as a landmark for the anteroposterior diameter (red bi-directional arrow). The craniocaudal diameter (blue bi-directional arrow) of the PT is parallel to the hyperechoic mucosal surface seen below the tonsil.

For transcervical US, the tongue and lingual tonsils are visualized using a curved low-frequency transducer placed on the suprahyoid region in a transverse plane. A swipe from the chin to towards the hyoid bone is performed (Figure 3a). The probe is then rotated 90 degrees clockwise to the sagittal plane and two consecutive midline-to-lateral swipes are performed for each side of the base of tongue (Figure 3b). Similarly, the palatine tonsils are visualized in two planes in the submandibular gland region (Figure 3c,d) [20]. The quality of both transoral and transcervical US scans will be rated using a 5-point Likert scale (1—Very poor; 2—Poor; 3—Fair; 4—Good; 5—Excellent) [35]. Patient-reported discomfort from transoral US will be quantified using an 11-point numeric rating scale from 0 (no discomfort) to 10 (worst discomfort imaginable).

#### 2.3.6. US Criteria for Tumor Detection

If an oropharyngeal tumor is clearly visualized via US examination, a “positive” result will be registered. If altered tonsillar appearance and/or doppler flow is visualized without a well-defined tumor and clinical evaluation cannot rule out a cancer, an “inconclusive” result is registered. If no suspected lesions are found—or benign lesions such as tonsilloliths [36], peritonsillar abscesses [24], or cysts [37] are visualized—thorough examination of both sides including normal Power Doppler examination is required to yield a “negative” result. We will register whether transoral US, transcervical US, or both techniques exams could detect an oropharyngeal tumor. Suspected tumor locations will be registered identically to the clinical registered categories (Section 2.3.2). The grade of oropharyngeal tumor suspicion will be further characterized using a 5-point Likert scale (1—Very low; 2—Low; 3—Intermediate.; 4—High; 5—Very high) [38]. 

#### 2.3.7. US Tumor Size Estimation and T-Staging

All US tumor measurements will be registered blinded to MRI and PET/CT. Small tumors will be measured transorally if they fit within the field-of-view of the transoral US transducers. In transverse transoral US of the palatine tonsils, the medial pterygoid muscle will serve as a lateral landmark (Figure 1c): the mediolateral diameter will be measured from the medial pterygoid muscle towards the tonsil’s medial surface. A perpendicular measurement will be performed for the anteroposterior diameter (Figure 1c). In sagittal view, the length of the tonsil tumor from the upper to lower pole will be measured as the craniocaudal diameter (Figure 1d). For the lingual tonsil, the hyoid bone will serve as a caudal landmark for the craniocaudal tumor diameter (Figure 2b). A perpendicular anteroposterior diameter will be measured through the tumor from the posterior surface of the lingual tonsil in the direction of the tongue apex. The mediolateral diameter is more difficult to measure transorally due to technical limitations of the US transducers. Instead, this diameter will be measured transcervically if the tumor can be visualized.

The greatest tumor diameters in three dimensions and an ultrasonographic T-stage will be registered considering tumor invasion of deep structures according to the UICC8 staging system. Transcervical US will be used to measure primary oropharyngeal tumors that are larger than the transoral US transducer’s field-of-view (Figure 3). We will register whether the transoral US or transcervical US was superior in terms of tumor measurement and invasion. 

### 2.4. Cross-Sectional Imaging (Comparison Tests)

All included patients with a clinical suspicion of OPSCC will receive a Gadolinium contrast-enhanced MRI within 1–2 weeks of the initial outpatient work-up. Included patients who do not receive an MRI—such as low clinical suspicion of OPSCC—will be excluded from the primary outcome analysis. FDG PET/CTs will be performed within 1–2 weeks of the initial outpatient work-up for a subgroup of patients with clinical unknown primary cancer or for therapeutic radiotherapy planning for patients with OPSCCs. An expert neuroradiologist (R.N.) will evaluate the contrast-enhanced MRIs while blinded to PET/CT, transoral and transcervical US, cytology, and histopathology results. Fused PET/CT scans will be evaluated by an expert nuclear medicine physician (D.D.) blinded to MRI, transoral and transcervical US, cytology, and histopathology results. Any neck CT scans performed due to diagnostic or therapeutic PET/CT will be separately analyzed by an oncoradiologist (J.M.H.) blinded to PET, MRI, transoral and transcervical US, cytology, and histopathology. 

For all cross-sectional imaging, scan quality will be rated using a 5-point Likert scale (1—Very poor to 5—Excellent quality) based on motion artifacts, dental artifacts, or missing contrast agents. For MRI and CT, the use of intravenous contrast agents will be reported. Tumor detection will be evaluated using the following criteria: positive (clearly visible oropharyngeal tumor), inconclusive (oropharyngeal asymmetry), and negative (no oropharyngeal tumor). A 5-point Likert score will also be reported for grading of oropharyngeal tumor suspicion (1—very low to 5—very high suspicion). 

**Figure 4:**
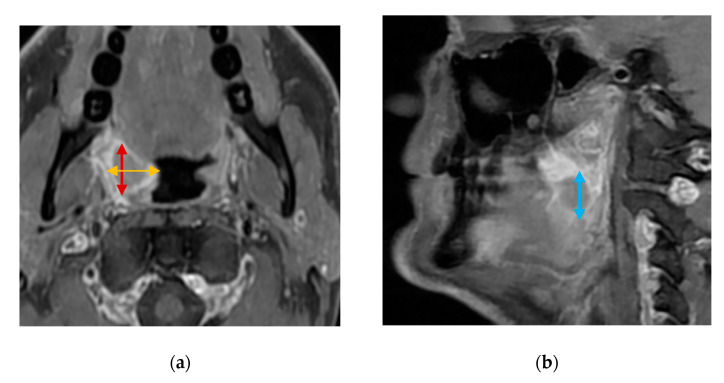
Contrast-enhanced, T1-weighted MRI images of a stage T1 squamous cell carcinoma of the right palatine tonsil: (**a**) axial orientation showing the mediolateral (yellow bi-directional arrows) and anteroposterior (red bi-directional arrows) diameters of the tumor; (**b**) sagittal orientation showing the craniocaudal (blue bi-directional arrows) diameter of the tumor.

CTs and MRIs will be evaluated for the greatest tumor diameters in three dimensions (craniocaudal, anteroposterior, and mediolateral) for visible tumors. Tumors will be measured in three perpendicular dimensions in axial (anteroposterior and mediolateral diameters) and coronal/sagittal planes (craniocaudal diameter) (Figure 4). A categorical T-stage will be estimated using CT and MRI according to the UICC8 staging system.

### 2.5. Reference Standard for Tumor Detection

The reference standard for tumor detection for all diagnostic tests will be the binary histopathology diagnosis (oropharyngeal tumor present vs. no oropharyngeal tumor). Medical charts of all included patients with an absence of oropharyngeal tumors after full examination will be looked up 3 months after concluding the study’s inclusion period to confirm that their initial benign diagnosis was correct. For patients with OPSCC, the final tumor location and T-stage will be defined via MDT conference. If primary tumors are resected, the pathologic T-stage and tumor size will be evaluated by an expert head and neck pathologist (T.K.A.). For all other primary tumors, the reference T-stage and tumor size will be determined via MDT, considering all available clinical and radiological findings. 

## 3. Outcome Definitions

### 3.1. Primary Outcomes

Sensitivity and specificity of USs (both transoral and transcervical) and MRI for the diagnostic work-up of patients with clinically suspected oropharyngeal tumors.

### 3.2. Secondary Outcomes

- Oropharyngeal tumor suspicion via US, MRI, PET/CT, and CT (5-point Likert scale).
- Detection of patients suspected to have unknown primary OPSCCs via transoral and transcervical US, MRI, and PET/CT.
- Tumor detection, size (mm) in three dimensions (craniocaudal, anteroposterior, and mediolateral), and volume (mm^3^) estimated clinically via US, MRI, CT, and histopathology.
- Categorical T-stage (Tx, T0, T1, T2, T3, or T4) measured clinically via US, MRI, and CT.

### 3.3. Other Outcomes

- Scan quality of transoral US, transcervical US, MRI, PET/CT, and CT (5-point Likert scale).
- Patient mouth opening ability (mm) and categorical trismus (>35 mm, ≤35 mm).
- An 11-point numeric rating scale for discomfort of oropharyngeal palpation and transoral US.
- Patient gagging severity index (5-point scale).
- Patient Mallampati score (grade I-IV).

## 4. Statistics

The full statistics plan can be seen in Supplementary Materials. The diagnostic accuracy of oropharyngeal primary tumor detection will be analyzed using the sensitivity, specificity, positive and negative predictive values, and overall accuracy of transoral and transcervical US, MRI, PET, and CT. The histopathology diagnosis and the final MDT specified tumor location will define true positive, false positive, true negative, and false negative results (Table 4). Positive and inconclusive diagnostic tests with correctly specified tumor locations will be analyzed as positive test results—due to the clinical consequence leading to further diagnostic interventions—if the reason for inconclusiveness is diagnostic uncertainty [39]. Non-diagnostic tests due to incomplete transoral US exams or missing contrast agents (MRI and CT) will be excluded from primary analysis, while a sub-analysis will include these cases. A multivariable logistic regression analysis will be performed using the reference standard diagnosis (oropharyngeal tumor vs. no oropharyngeal tumor) as the binary outcome variable, while results of US or cross-sectional imaging, time between scans, and the study center will be included as covariables. Generalized estimating equations (GEE) will account for correlations within the data, with an exchangeable correlation structure.

A sub-analysis of the OPSCC detection rate will be compared between transoral US, transcervical US, and MRI stratified by final MDT T-stage. Another sub-analysis will be conducted for OPSCC detection stratified by tumor location (palatine tonsil, lingual tonsil, overlapping palatine/lingual tonsils, and other oropharynx). A subgroup analysis will be performed on patients with clinically suspected unknown primary cancer, where narrowband imaging, transoral US, transcervical US, MRI, CT, and PET/CT will be compared in terms of diagnostic accuracy for primary oropharyngeal tumor detection. McNemar’s test will be used to determine whether there are statistically significant differences between test sensitivities. To assess for a possible learning curve in performing transoral and transcervical US, we will perform an additional sub-analysis: each ultrasonographer’s included patients will be divided into two equal halves based on the inclusion date. The diagnostic accuracy per ultrasonographer will be compared between the first and second half of included patients. 

For tumor size correlation, the greatest tumor diameters in craniocaudal, anteroposterior, and mediolateral diameters and tumor volumes will be compared between US and cross-sectional imaging using scatter plots, Pearson’s correlation coefficient (r), and Bland–Altman plots [40]. For patients with surgically resected tumors, a sub-analysis will be performed comparing pre-operative US and cross-sectional imaging to the formalin-fixated histopathology’s greatest tumor diameter. 

To account for possible selection bias, all screened patients from the date of first patient inclusion at each center will be prospectively evaluated for potential eligibility according to our inclusion criteria. Eligible patients that were not included due to logistical reasons will be compared to included patients using descriptive statistics and Pearson’s Chi^2^ test for categorical variables stated in Table 1. We will also compare the age of these two groups using an unpaired *t*-test. We will also compare the inclusion rate of eligible patients between the three centers.

RStudio statistics software version 4.1.0 will be used to perform all analyses [41], 95% confidence intervals (95%CI) will be reported for all measures of diagnostic accuracy, and statistical significance will be reported as *p*-values < 0.05. 

## 5. Sample Size Calculation

A sample size calculation was performed based on a feasibility study we conducted on 26 patients that received oropharyngeal US and MRI [26]. This study resulted in a 3.8% and 15.4% rate of discordance between US and MRI, respectively, in terms of successfully classifying patients according to their final diagnosis. Using a power of 90% and an alpha of 5%, we conducted a power calculation using the following online calculator (accessed on 1 January 2023): http://powerandsamplesize.com/Calculators/Compare-Paired-Proportions/McNemar-Z-test-2-Sided-Equality. To account for the exclusion of non-diagnostic tests, such as incomplete transoral US or missing intravenous contrasts for MRI, an additional 10% will be added to the sample size. The minimum required sample size will, therefore, be 161 patients referred with clinically suspected oropharyngeal cancer or unknown primary cancer. We expect to have concluded inclusion within 1½ years. Any additional patients included prior to benign histopathology or lymphoma diagnoses that do not routinely receive MRI scans will be used for subgroup analysis. 

## 6. Discussion

The intent of this protocol is to be transparent with our systematic transoral and transcervical US method for this multicenter prospective study of diagnostic accuracy blinded to histopathology and cross-sectional imaging.

First-time patient referrals for cancer work-up with a suspicion of OPSCC or unknown primary cancer will be offered inclusion. This will result in a cohort of patients of different T-stages, benign and malignant diagnoses, and multiple histopathological diagnoses. The sample cannot be generalized to the general population since patients referred for cancer work-up are already a highly selected patient population. Instead, our oropharyngeal tumor detection analyses will be representative for head and neck cancer centers with first-time referrals. Prior studies utilizing transcervical US are limited by selection bias regarding the analysis of detection sensitivities, since patients already have histopathologically verified OPSCCs at the time of inclusion or previously available cross-sectional imaging [16,17,42]. Our study will instead present unbiased tumor detection sensitivity and specificity. 

The greatest limitations for both transoral and transcervical US include the learning curve required to both perform and interpret the imaging. For our study, all study investigators that include patients will undergo an introductory course in transoral and transcervical US of the oropharynx and regularly receive supervision from the primary investigator (M.G-Z.). Furthermore, a sub-analysis will be performed comparing the tumor detection rates by stratifying US tumor detection sensitivity and specificity into “inexperienced” and “experienced” ultrasonographer groups. 

Secondarily, if tumor size or T-staging is comparable with cross-sectional imaging, this will provide head and neck surgeons with a point-of-care tool to evaluate the primary tumor extent. Treatment decisions, such as operability versus chemoradiation, can then be made at the time of the patient’s first visit. One other limitation is that cross-sectional imaging is not a true gold standard for evaluating tumor size but histopathologic examination of the entire tumor specimen is. In practice, most patients with OPSCC are treated using chemoradiation, while a subset of patients with small primary tumors are operated upon. Considering this limitation, US can potentially be more precise at measuring tumor size than cross-sectional imaging. To explore this, we will perform a subgroup analysis on patients who are offered surgical treatment to compare histopathologic tumor size with cross-sectional imaging and US.

In conclusion, this study protocol provides a systematic approach to the transoral US technique of the oropharynx, which has not been well described in the current literature. This will provide surgeons and/or radiologists with a new diagnostic modality that may improve the diagnostic work-up for patients with oropharynx cancer.

## Figures and Tables

**Table 1:** Screening log example for the SPOT-US trial.

Screen ID	Age	Sex	Clinical Diagnosis	Offered Inclusion	Reasons Not Included	Excluded	Reasons for Exclusion	Final Histopathology Diagnosis	Final Tumor Location	Final T-Stage
			Suspected oropharynx tumor or unknown primary cancer	Yes, No	Declined, Missed, Other	Yes, No	Inclusion/exclusion criteria not met	No tumor, Squamous cell carcinoma, Benign tumor, Lymphoma, Other	Palatine tonsil, Lingual tonsil, Other oropharynx, Unknown, Other head and neck tumor.	Tx, T0, T1, T2, T3, T4

**Table 2:** Eligibility criteria during initial clinical work-up of referred patients with suspicion of oropharyngeal cancer.

Criteria	Description
Inclusion	Clinically visible or palpable oropharyngeal tumor. Visible asymmetry with palpable firmness in the palatine and/or lingual tonsils. Suspected squamous cell carcinoma metastasis in neck levels II–IV with no visible or palpable primary tumor.
Exclusion	Prior oropharynx cancer or head and neck radiotherapy. Prior MRI or PET/CT within three months of inclusion. Unable to understand the written or oral study information.

**Table 4:** Definition of true positive (TP), false positive (FP), false negative (FN), and true negative (TN) from histopathology diagnosis and acronym MDT specified tumor location.

Test Result	Oropharyngeal Tumor	No Oropharyngeal Tumor
Positive or inconclusive, correct location	TP	FP
Positive or inconclusive, wrong location	FN	FP
Negative	FN	TN

## Data Availability

No new data were created or analyzed in this study. Data sharing is not applicable to this article.

## Supplementary Materials

The following supporting information can be downloaded at: https://www.mdpi.com/article/10.3390/diagnostics14060577/s1, Statistical Analysis Plan (SAP). Figure S1: Bland-Altman Plot; Table S1: Test results 2 × 3 table; Table S2: Long dataset format; Table S3: GEE logistic regression.

## Abbreviations

95%CI	95% Confidence interval	MPM	Medial pterygoid muscle
BOT	Base of tongue	MRI	Magnetic resonance imaging
CT	Computerized tomography	OPSCC	Oropharyngeal squamous cell carcinoma
FDG	18F-Fluorodeoxyglucose	PET/CT	Positron emission tomography/computerized tomography
GEE	Generalized estimating equations	PT	Palatine tonsil
GGM	Genioglossus muscle	SGM	Styloglossus muscle
GHM	Geniohyoid muscle	SMG	Submandibular gland
HPV	Human papillomavirus	SPM	Stylopharyngeus muscle
ICA	Internal carotid artery	SPOT-US	Surgeon-Performed Oropharyngeal Transoral UltraSonography
ITM	Intrinsic tongue muscles	UICC8	*Union for International Cancer Control 8* *th Edition*
MDT	Multidisciplinary team	US	Ultrasonography
