# Supplementary material for: Comparison of Transoral and Transcervical Ultrasonography with MRI for the Diagnostic Work-Up of Oropharynx Tumors: A Protocol for a Multicenter Clinical Trial (SPOTUS)
Source: Diagnostics (Basel). 2024 Mar 8;14(6):577. doi: 10.3390/diagnostics14060577 (PMC10968743; doi:10.3390/diagnostics14060577)
Supplement: Supplementary file 1 [file diagnostics-14-00577-s001.zip › diagnostics-2901202-supplementary.pdf]

Statistical Analysis Plan (SAP)

Title: Comparison of Transoral and Transcervical Ultrasound with MRI for the diagnostic workup of oropharynx tumors – a protocol for a multicenter clinical trial (SPOTUS)

Trial registration number (clinicaltrials.gov): NCT05696314.

Roles and responsibilities:
Professor Annette Kjær Ersbøll, Ph.D.

- Role: senior statistician.
- Responsibilities: revision and approval of SAP.

Martin Garset-Zamani, MD, Ph.D.-scholar

- Role: primary investigator.
- Responsibilities: defining research questions, outcome measures and writing of SAP.

Associate Professor Tobias Todsen, MD, Ph.D

- Role: chief investigator.
- Responsibilities: defining research questions and outcome measures.

This SAP is structured according to recommendations from "Guidelines for the Content of Statistical Analysis Plans in Clinical Trials." by Gamble C, Krishan A, Stocken D, Lewis S, Juszczak E, Doré C, et al. published in JAMA 2017;318:2337-43.

Contents

1. Introduction....4

1.1 Background....4

1.2 Research question....4

1.3 Hypotheses....4

2. Methods....5

2.1 Study design....5

2.2 Randomization....5

2.3 Sample size....5

2.4 Framework....6

2.5 Statistical interim analyses and stopping guidance....6

2.6 Timing of final analysis....6

2.7 Timing of outcome assessments....6

3. Statistical principles....7

3.1 Confidence intervals and P values....7

3.2 Adherence and protocol deviations....7

3.2.1 Definition of adherence to the intervention....7

3.2.2 Description of adherence....7

3.2.3 Definition of protocol deviations for the trial....7

3.2.4 Description of which protocol deviations will be summarized....7

3.3 Analysis populations....7

4. Trial population....8

4.1 Screening data....8

4.2 Eligibility....8

- **Prior oropharynx cancer or head and neck radiotherapy. 8**
- **Prior MRI or PET/CT within three months of inclusion. 8**

• Unable to understand the written or oral study information. 8

4.3 Recruitment 8

4.4 Withdrawal/follow-up 9

4.5 Baseline patient characteristics 9

5. Analysis 10

5.1 Outcome definitions 10

5.1.1 Primary outcomes 10
5.1.2 Secondary outcomes 10
5.1.3 Other outcomes 10

5.2 Analysis methods 10
5.2.1 Primary oropharynx tumor detection reference standard 10
5.2.2 Definition of test results 10
5.2.3 Diagnostic accuracy of primary oropharynx tumor detection within patients 11
5.2.4 Multivariable logistic regression for correct location-specific tumor detection 12
5.2.5 Comparing tumor size estimation between SPOTUS and MRI 12
5.2.6 Comparing tumor volume estimation between SPOTUS and MRI 13
5.2.7 Patient reported discomfort 13

5.3 Missing data 13

5.4 Additional analyses 14

Additional sub-analyses of OPSCC detection sensitivity will be compared between transoral US, transcervical US and MRI stratified by: 14

A) Final MDT T-stage 14

B) Final tumor location (palatine tonsil, lingual tonsil, other oropharynx). 14

To assess for a possible learning curve in performing transoral and transcervical US, the diagnostic accuracy per ultrasonographer will be compared between the first and second half of included patients. Sensitivity and specificity analyses will be repeated for the resulting two groups and compared with the McNemar's test. 14

5.5 Harms 14

5.6 Statistical software 14

References 14

1. Introduction

1.1 Background

Current clinical examination and diagnostic imaging for small oropharynx squamous cell carcinoma (OPSCC) is insufficient for rapid diagnosis. High-frequency, small-footprint transoral ultrasound (US) may provide greater sensitivity while also improving specificity compared to current diagnostic imaging with Magnetic Resonance Imaging (MRI), Computerized Tomography (CT), and 18F-Fluorodeoxyglucose (FDG) Positron Emission Tomography/CT (PET/CT).

1.2 Research question

In patients referred to tertiary head and neck cancer centers with suspicion of OPSCC, what is the diagnostic accuracy of outpatient-performed transoral and transcervical US compared to MRI for detecting and staging oropharyngeal tumors?

1.3 Hypotheses

1. Transoral US has greater sensitivity compared to MRI and PET/CT for detecting primary tumors in patients with OPSCCs smaller than 20mm (stage T1).
2. Transoral US has greater specificity compared to MRI in patients without oropharynx cancer.
3. Transoral US has greater sensitivity and specificity for OPSCC detection compared to transcervical US due to increased superficial tissue resolution.
4. Combined transoral and transcervical US examination can act as a surrogate to MRI for estimating primary tumor size and T-stage.

2. Methods

2.1 Study design

This is a prospective, multicenter non-randomized clinical trial exploring the use of transoral and transcervical US in the outpatient clinic for detecting oropharyngeal tumors blinded to reference standard imaging and histopathology (Clinicaltrials.gov registration NCT05696314). The trial design will be a single group paired diagnostic study comparing transoral and transcervical US to MRI and PET/CT. The aim of the trial is to explore whether US is superior to current diagnostic imaging in detection of small primary OPSCCs. The trial will be performed in the departments of Otorhino-laryngology, Head & Neck Surgery in three public hospitals in Denmark at Copenhagen University Hospital - Rigshospitalet, Aarhus University Hospital, and Zealand University Hospital - Køge. In Denmark, patients suspected of having cancer are entitled to a free diagnostic work-up and treatment at public hospitals, funded by the national tax system.

2.2 Randomization

The study is not randomized.

2.3 Sample size

A sample size calculation was performed based on a feasibility study we conducted on 26 patients which received oropharyngeal US and MRI [26]. This study resulted in a 3.8% and 15.4% rate of discordance between US and MRI, respectively, in terms of success-fully classifying patients according to final diagnosis. Using a power of 90% and an alpha of 5%, we conducted a power calculation using the following online calculator: http://powerandsamplesize.com/Calculators/Compare-Paired-Proportions/McNemar-Z-test-2-Sided-Equality. To account for exclusion of non-diagnostic tests, such as incomplete transoral US or missing intravenous contrast for MRI, an additional 10% will be added to the sample size. The

minimum required sample size will therefore be 161 patients referred with clinical suspected oropharyngeal cancer or unknown primary. We expect to have concluded inclusion within 1½ years. Any additional patients included prior to benign histopathology or lymphoma diagnoses that do not routinely receive MRI scans will be used for subgroup analysis.

2.4 Framework

We will test whether the tumor detectability with the intervention (transoral and transcervical US of the oropharynx) is significantly different from the comparison tests (MRI, CT and/or PET/CT).

2.5 Statistical interim analyses and stopping guidance

Interim analysis will be performed on included patients to assess for availability of MRIs and that the final diagnosis has been established. We aim to include roughly 2:1 cases in terms of oropharyngeal tumors and benign patients without oropharyngeal tumors. This is to ensure that we have a large sample size of oropharyngeal tumors for tumor size correlation between US and MRI for the secondary outcomes.

2.6 Timing of final analysis

All analyses will be performed once the recruitment period has concluded, and the final included patient has completed their diagnostic work-up appointments, no later than 3 months after inclusion of the final patient.

2.7 Timing of outcome assessments

Outcome assessments will be made prospectively. Transoral and transcervical US results will thus be registered at the time of inclusion. MRI and PET/CTs will be rated prospectively after acquisition of the scans and prior to analysis.

3. Statistical principles

3.1 Confidence intervals and P values

Reported P-values will be regarded as significant if $p < 0.05$. 95% confidence intervals (95% CI) will also be reported.

3.2 Adherence and protocol deviations

3.2.1 Definition of adherence to the intervention

Adherence will be defined as a patient with the following available data: Danish personal identification number (CPR), outpatient transoral/transcervical US, MRI, and final diagnosis.

3.2.2 Description of adherence

The adherence will be visualized in an inclusion flow chart with number of included patients minus the number of drop-outs due to missing adherence data.

3.2.3 Definition of protocol deviations for the trial

Patients will be excluded from analysis if any of the adherence data is missing.

3.2.4 Description of which protocol deviations will be summarized

As specified above, the number of patients excluded will be summarized in the inclusion flow chart.

3.3 Analysis populations

A complete case analysis will be used for each comparison. All included patients will have available US and MRI, but only a subgroup will have available PET/CT. Thus, the analyses performed on this subgroup will be separate. Another subgroup of included patients without available MRI will be analyzed separately.

4. Trial population

4.1 Screening data

Patients referred for fast-track cancer work-up to the head & neck surgical departments at Copenhagen University Hospital – Rigshospitalet, Zealand University Hospital – Køge and Aarhus University Hospital, with suspected OPSCC or unknown primary cancers will be screened for eligibility.

4.2 Eligibility

During patients' initial work-up at the above hospital departments, the following criteria will be evaluated and patients meeting the criteria will be invited to participate in the study:

Inclusion criteria:

- Clinically visible or palpable oropharyngeal tumor.
- Clinical asymmetry in the palatine and lingual tonsils.
- Suspected or confirmed squamous cell carcinoma metastasis in neck levels II-IV with no visible or palpable primary tumor.

Exclusion criteria:

- Prior oropharynx cancer or head and neck radiotherapy.
- Prior MRI or PET/CT within three months of inclusion.
- Unable to understand the written or oral study information.

4.3 Recruitment

Patients will be recruited at the time of their initial work-up, where they will be offered the intervention consisting of US of the oropharynx.

A flow diagram will summarize the number of screened patients, number of eligible patients, number of ineligible patients, number of patients included, and number of patients excluded. Reasons for ineligibility and exclusion will be summarized.

4.4 Withdrawal/follow-up

Results from the interventions are registered prospectively at the patient's first visit, thus no follow-up appointments are required.

4.5 Baseline patient characteristics

Baseline characteristics for included patients and non-included eligible patients will be presented in a descriptive table and figures, including:

- Demographic data:
  - Age (numerical)
  - Sex (categorical: male, female)
- Clinical diagnosis:
  - Suspected oropharyngeal tumor (categorical: palatine tonsil, lingual tonsil, other oropharynx, unknown primary).
- Final diagnosis:
  - Oropharyngeal tumor: (categorical: yes, no)
  - Oropharyngeal squamous cell carcinoma: (categorical: yes, no)
  - Tumor location: (categorical: palatine tonsil, lingual tonsil, other oropharynx, unknown primary)
  - T-stage: (categorical: Tx, T0, T1, T2, T3, T4)

Categorical data will be reported as frequencies and proportions, while continuous data will be summarized with means and standard deviations (or median and interquartile ranges if non-

normally distributed). Differences in categorical and continuous data will be assessed with Pearson's Chi^2 and Student's t-test.

5. Analysis

5.1 Outcome definitions

5.1.1 Primary outcomes

- Detection of oropharyngeal tumors by transoral and transcervical US in the correct oropharyngeal location.
- Detection of oropharyngeal tumors by MRI in the correct oropharyngeal location.

5.1.2 Secondary outcomes

- Oropharyngeal tumor suspicion by US, MRI, PET/CT, and CT (5-point Likert scale).
- Detection of unknown primary OPSCCs by transoral and transcervical US and PET/CT in the correct oropharyngeal location.
- Detection of oropharyngeal tumors by contrast-enhanced CT in the correct oropharyngeal location.
- Tumor size (mm) in three perpendicular dimensions (craniocaudal, anteroposterior, mediolateral) estimated clinically, by US, MRI, CT, and histopathology.
- Tumor volume (mm3) estimated clinically, by US, MRI, and CT.
- Categorical T-stage (T1, T2, T3, or T4) measured clinically, by US, MRI, and CT.

5.1.3 Other outcomes

- Scan quality of transoral US, transcervical US, MRI, PET/CT, and CT (5-point Likert scale).
- Patient mouth opening (mm) and categorical trismus (>35mm, <35 mm).
- 11-point Numeric Rating Scale for discomfort of oropharyngeal palpation and tran-soral US.
- Patient gagging severity index (5-point scale).
- Patient Mallampati score (grade I-IV).

5.2 Analysis methods

5.2.1 Primary oropharynx tumor detection reference standard

The reference standard will be a final diagnosis, based on either a confirmatory biopsy, resection, or follow-up (if no biopsy is available). The outcome will be specified as "oropharyngeal tumor" or "no oropharyngeal tumor".

5.2.2 Definition of test results

Test results from US, MRI, CT, and PET/CT will be reported as:

- Positive (clearly visualized primary tumor)
- Inconclusive (asymmetry without visible focal tumor)
- Negative (no suspicion of oropharyngeal tumor)

These results will be displayed in a descriptive 2x3 table comparing with the reference standard final diagnosis.

5.2.3 Diagnostic accuracy of primary oropharynx tumor detection within patients

US, MRI, CT, and PET/CT test results will be defined as true positive (TP), true negative (TN), false positive (FP) and false negative (FN) in reference to the final diagnosis and tumor location. Overall diagnostic accuracy measures will be calculated based on the test results, while adjusting for concordance of tumor location between the test and the actual tumor location.

Measures of diagnostic accuracy used include: sensitivity (Se), specificity (Sp), positive predictive values (PPV), and negative predictive values (NPV), and overall accuracy (Acc) calculated using the following formulae:

- Se = TP / (TP+FN)
- Sp = TN / (TN+FP)
- PPV = TP / (TP+FP)
- NPV = TN / (TN+FN)
- Acc = (TP + TN) / (TP + TN + FP + FN)

We will exclude non-diagnostic tests due to incomplete transoral US exams or missing contrast agents (MRI and CT) from primary analysis while a sub-analysis will include these cases. Inconclusive test results due to diagnostic uncertainty but with correctly specified tumor locations will be analyzed as a positive result due to clinical consequences often leading to further biopsies, or tonsillectomy [1]. (Supplementary Table S1) A supplementary sensitivity, positive predictive value, and overall accuracy analyses will be performed excluding inconclusive cases.

5.2.4 Multivariable logistic regression for correct location-specific tumor detection

A repeated measures dataset format will be used, with each patient represented individual rows of data per test method (US, MRI, CT, and PET/CT) (Supplementary Table S2). Two multivariable logistic regression models with Generalized Estimating Equations (GEE) will be performed accounting for the correlation between observations within patients. An exchangeable correlation structure will be used. The models will be performed with complete data only, thus model 1 will consist of all patients with available US and MRI, while model 2 will consist of patients with available US, MRI, CT, and PET/CT. For both models, the outcome variable will be indicated as a "correct test result" variable (correct = 1, incorrect = 0). Each imaging tests outcomes will be computed by combining the tumor detection results (TP or TN). A covariable "test method" will be the primary covariable of interest, where MRI will be set as reference. A "time between tests" covariable with continuous data will refer to the days between US and MRI, CT, and/or PET/CT are performed, where US will be the reference. The "time between variables" will be checked for linearity, and if non-linear, a categorical time variable will be used instead. Odds ratios, 95% confidence intervals and p-values will be reported. Significance level will be <0.05 (Supplementary Table S3).

5.2.5 Comparing tumor size estimation between SPOTUS and MRI

Scatter plots will illustrate the associations between greatest tumor diameter estimated with US vs. MRI and CT. Pearson's correlation coefficients will be calculated. To test for difference, paired t-tests will be performed. Bland-Altman plots will be performed comparing the mean differences and limits of agreement (LoA) between US-MRI, US-CT, or CT-MRI measurements for the greatest tumor size and tumor volumes [2]. LoA will be calculated as mean difference +/- 2 standard deviations (Supplementary Figure S1).

A sub-analysis will be performed stratifying cases into four groups based on final clinical and radiographic T-stage as reference. In patients with available tumor resections, a sub-analysis will be performed using the histopathologic greatest tumor diameter as the gold reference standard.

Agreement between categorical T-stage (T1, T2, T3, T4) between US and MRI will be performed with 4x4 tables. The percentage agreement and weighted Cohen's Kappa values will be calculated.

5.2.6 Comparing tumor volume estimation between SPOTUS and MRI

Tumor volume will be estimated by measuring three perpendicular greatest tumor diameters in three dimensions and using the volume formula for an ellipse: Volume = 4/3 * π * (length / 2) * (width / 2) * (height / 2). Scatter plots, Bland-Altman plots, Pearson's correlation, and paired t-tests will be performed comparing US vs. MRI. A sub-analysis will be performed stratifying cases based on final T-stage.

5.2.7 Patient reported discomfort

11-point numeric rating scales for patient reported discomfort from transoral US will be compared to clinical examination with transoral palpation of the tonsils and tongue base [3]. If the data is normally distributed, the mean discomfort will be reported and compared using a paired t-test. If data is not normally distributed, median values and non-parametric testing will be performed.

5.3 Missing data

Included patients with missing MRI scans, or confirmatory biopsy results (if cancer is suspected) will be reported as missing data and excluded from final analysis.

5.4 Additional analyses

Additional sub-analyses of OPSCC detection sensitivity will be compared between transoral US, transcervical US and MRI stratified by:

A) Final MDT T-stage

B) Final tumor location (palatine tonsil, lingual tonsil, other oropharynx).

To assess for a possible learning curve in performing transoral and transcervical US, the diagnostic accuracy per ultrasonographer will be compared between the first and second half of included patients. Sensitivity and specificity analyses will be repeated for the resulting two groups and compared with the McNemar's test.

5.5 Harms

Adverse events during the intervention, such as bleeding from the oropharynx, or vomiting, will be registered.

5.6 Statistical software

RStudio statistics software version 4.1.0 will be used to perform all analyses [4].

References

1. Shinkins, B.; Thompson, M.; Mallett, S.; Perera, R. Diagnostic accuracy studies: how to report and analyse inconclusive test results. *BMJ* **2013**, *346*, f2778-f2778, doi:10.1136/bmj.f2778.
2. Bland, J.M.; Altman, D.G. Statistical methods for assessing agreement between two methods of clinical measurement. *The Lancet* **1986**, *327*, 307-310.
3. Todsen, T.; Tolsgaard, M.G.; Benfield, T.; Folke, F.; Jakobsen, K.K.; Gredal, N.T.; Ersbøll, A.K.; Von Buchwald, C.; Kirkby, N. Higher SARS-CoV-2 detection of oropharyngeal compared with nasopharyngeal or saliva specimen for molecular testing: a multicentre randomised comparative accuracy study. *Thorax* **2023**, *78*, 1028-1034, doi:10.1136/thorax-2022-219599.
4. *R Core Team (2020). R: A language and environment for statistical computing. R Foundation for Statistical Computing, Vienna, Austria.*

Supplementary Table S1: Test results 2x3 table

		Reference standard	
		Cancer	Benign
Test	Positive	TP	FP
	Inconclusive	TP[§]/FN[#]	FP[§]/TN[#]
	Negative	FN	TN

Legend: 2x3 contingency table illustrating comparison of test results (outpatient transoral and transcervical ultrasound, MRI, and PET/CT) with the reference standard (MDT diagnosis based on clinical and histopathology results, or clinical follow-up up to 3 months for benign cases). Inconclusive results can be either excluded, analyzed as positive results, or as negative results, based on the clinical context. In our study, a missed cancer diagnosis is a serious consequence of an inconclusive test, so these results will be handled as positive results. This results in increased sensitivity but reduced specificity with increasing numbers of inconclusive cases.

TP = true positive, FP = false positive, FN = false negative, TN = true negative, § = if inconclusives are analyzed as positive tests, # = if inconclusives are analyzed as negative tests.

Supplementary Table S2: Long dataset format

Case ID	Reference diagnosis (0=benign, 1=cancer)	Reference tumor location (0=no tumor, 1=tonsil, 2=tongue base)*	Test method	Test detection result (0=negative, 1=positive, 2=inconclusive)	Merged test detection result (0=negative, 1=positive/inconclusive)	Test tumor location (0=no tumor, 1=tonsil, 2=tongue base)	Time between tests	Test correct (0=incorrect, 1=correct)
1	1	1	Ultrasound	1	1	1	0	1
1	1	1	MRI	2	1	1	7	1
1	1	1	PET/CT	1	1	1	9	1
2	0	0	Ultrasound	0	0	0	0	1
2	0	0	MRI	2	1	1	6	0
3	1	2	Ultrasound	1	1	2	0	1
3	1	2	MRI	1	1	1	7	0

1 **Legend:** *For simplicity, this variable is limited to fewer tumor locations than the actual variable used in the protocol.

Supplementary Table S3: GEE logistic regression

Variable	Units	Odds ratio	95% Confidence Interval	P-value
Test method	MRI (reference)			
	PET/CT			
	Ultrasound			
Time between tests (days)				
Study Center	Rigshospitalet (reference)			
	Køge			
	Århus			

Legend: result table from multivariable logistic regression with generalized estimating equations. Outcome variable is a correct test result, defined as a true positive or true negative test coupled with the correct tumor location specified by the test in reference to the final multidisciplinary team diagnosis.

Supplementary Figure S1: Bland-Altman Plot

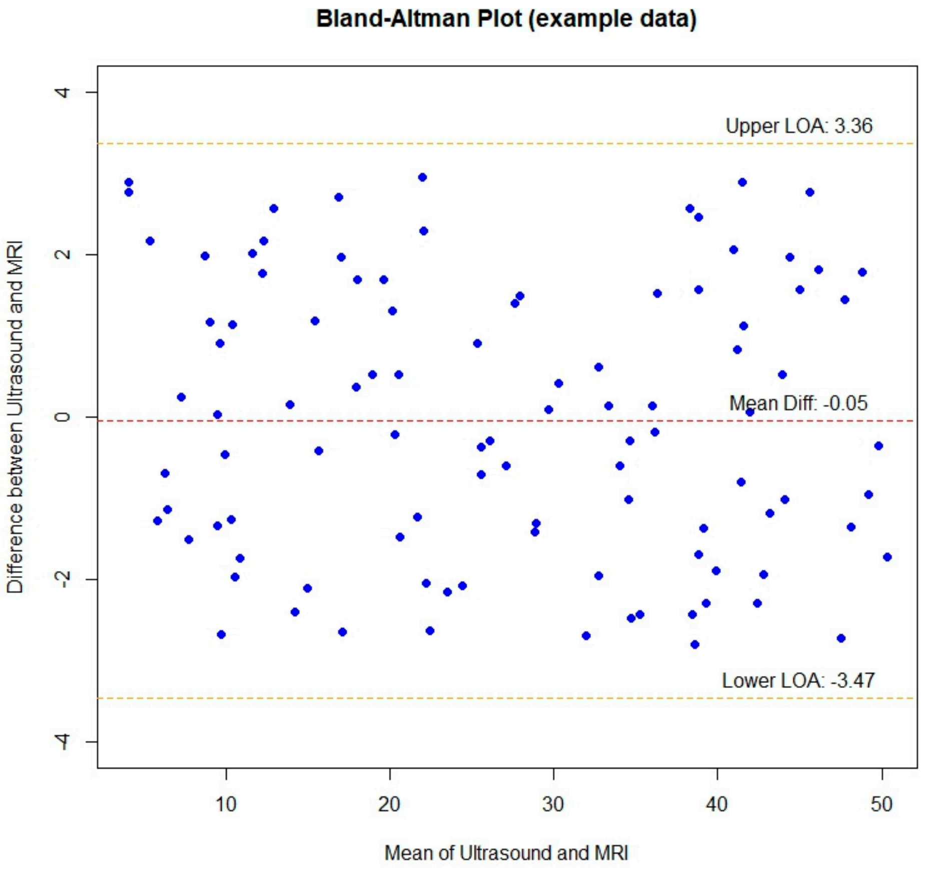

Legend: Example data illustrating a Bland-Altman plot comparing the differences between ultrasound and MRI. *LOA = limit of agreement.*
